# Underscoring the effect of swab type, workflow, and positive sample order on swab pooling for COVID-19 surveillance testing

**DOI:** 10.1038/s41598-023-34337-y

**Published:** 2023-05-03

**Authors:** Maxwell J. Kalinowski, Devon R. Hartigan, Neal M. Lojek, Bryan O. Buchholz, Chiara E. Ghezzi

**Affiliations:** Department of Biomedical Engineering, University of Massachusetts-Lowell, 1 University Avenue, Lowell, MA 01854 USA

**Keywords:** Biomedical engineering, Population screening

## Abstract

Sample pooling is a promising strategy to facilitate COVID-19 surveillance testing for a larger population in comparison to individual single testing due to resource and time constraints. Increased surveillance testing capacity will reduce the likelihood of outbreaks as the general population is returning to work, school, and other gatherings. We have analyzed the impact of three variables on the effectiveness of pooling test samples: swab type, workflow, and positive sample order. We investigated the performance of several commercially available swabs (Steripack polyester flocked, Puritan nylon flocked, Puritan foam) in comparison to a new injected molded design (Yukon). The bench-top performance of collection swab was conducted with a previously developed anterior nasal cavity tissue model, based on a silk-glycerol sponge to mimic soft tissue mechanics and saturated with a physiologically relevant synthetic nasal fluid spiked with heat-inactivated SARS-CoV-2. Overall, we demonstrated statistically significant differences in performance across the different swab types. A characterization of individual swab uptake (gravimetric analysis) and FITC microparticle release suggests that differences in absorbance and retention drive the observed differences in Ct of the pooled samples. We also proposed two distinct pooling workflows to encompass different community collection modes and analyzed the difference in resulting positive pools as an effect of workflow, swab type, and positive sample order. Overall, swab types with lower volume retention resulted in reduced false negative occurrence, also observed for collection workflows with limited incubation times. Concurrently, positive sample order did have a significant impact on pooling test outcome, particularly in the case of swab type with great volume retention. We demonstrated that the variables investigated here affect the results of pooled COVID-19 testing, and therefore should be considered while designing pooled surveillance testing.

## Introduction

As the COVID-19 pandemic persists, testing is paramount to prevent potential virus outbreaks while resuming work, school, and social activities. Testing is a powerful management tool, but extensive mass surveillance screening requires new testing strategies. To transition back to in-person gathering and working, widespread screening initiatives need to be accessible, accurate, and affordable. The rate of screening is limited by time and the availability of diagnostic resources, so individual surveillance testing cannot always be a viable strategy. A developing solution to these limitations is pooling test samples. According to the Centers for Disease Control and Prevention (CDC), pooling consists of combining specimens from several individuals into one pool and conducting a diagnostic analysis, in this case, to detect SARS-CoV-2^1^. Using this approach, the cost of COVID-19 screening can be dramatically reduced to almost one tenth of individual testing depending on the number of samples in the pool. In addition to reducing laboratory workloads and reducing expenses, pooling provides a more manageable method for screening large groups such as in schools and workplaces^2^.

Early pooling efforts employed a workflow that is best described as a combination of aliquots or sample pooling^3^. Two samples are collected from each patient: one for combination in the pool (the “pool” sample) and one for deconvolution if the pool tests positive (the “archive” sample)^4^. Both samples are placed into separate vials of transport media (Viral Transport Media, Universal Transport Media, saline etc.) and an aliquot from each sample is combined into one vial, and the resulting pooled sample is tested. If there is a combination of positive and negative samples in the pool, the positive samples will be diluted by the negative samples, possibly reducing the viral concentration below the positive threshold and, potentially increasing the likelihood of a false negative^1^. More recent studies have eliminated the issue of dilution by immediately combining swabs in the same media at the time of collection (swab pooling) instead of combining aliquots in the laboratory, having been shown to retain a sensitivity of 99%^3^. Additionally, the swab pooling method dramatically reduces the number of collection tubes, storage space, as well as handling of and potential exposure to positive patient samples for laboratory personnel.

Pooling shows promise to drastically increase screening capacity. However, our collective understanding of which variables affect pooling outcome and how their relative weights is still in its infancy^5^. In this work, we aim to parametrize pooling approaches to optimize its use as a screening strategy. The experimental variables analyzed here include swab type, collection media volume, type of workflow, pool size, and positive sample order (positive first or positive last). We begin by describing two distinct workflows: dip and discard (DDW) and combine and cap (CCW). All studies were conducted using a previously developed^6,7^ bench top in vitro experimental model of the nasal cavity comprised of silicone tubing lined with a silk-glycerol sponge. The sponge mimics the soft tissue of the nasal cavity, and the geometry of this model enables clinically relevant swabbing in a preclinical setting. The model also incorporates an artificial nasal fluid to mimic the viscosity and conditions of sample collection. Use of this model to isolate swabbing variables before advancing to clinical trials will save time, money, and valuable clinical samples. There are significant differences in sample uptake and release across swab types, thus, we describe these differences between four main swab types (polyester and nylon flocked, foam, and injection molded) through gravimetric analysis for mass uptake and particle release quantification to mimic cellular release. Lastly, we explore the relationship between workflow, positive sample order, and sample cycle threshold (Ct) value by spiking the artificial nasal fluid with heat inactivated SARS-CoV-2 virus and conducting Quantitative Reverse Transcription Polymerase Chain Reaction (RT-qPCR). The observed difference in Ct between workflows and positive sample orders is dependent on the type of swab used. Injection molded (IM) swabs appear to perform most consistently across all variables due to its relatively low volume retention and high release capacity. This data suggests that these variables are important to consider in validation studies when designing pooled screenings for COVID-19.

## Results

### Single swab pick up and release quantification

**Figure 1:**
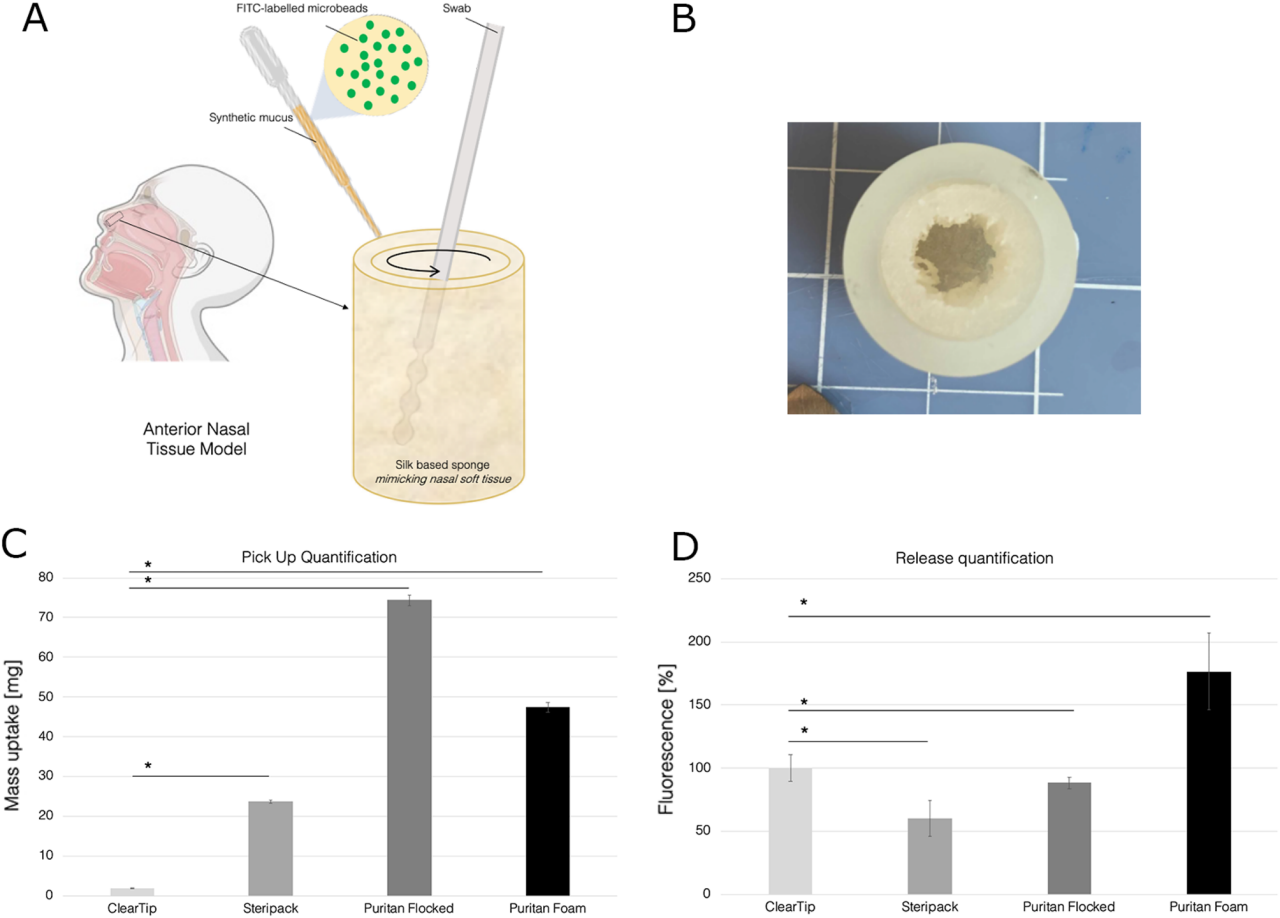
Swab characterizations. (**A**) Anterior nasal tissue model comprising a silk-based sponge loaded with synthetic nasal mucus spiked with FITC-labelled microbeads, as a surrogate for cellular material. (**B**) Macro image of the silk-based anterior nasal tissue model. Scale bar = 10 mm. (**C**) Mass uptake during the benchtop swabbing procedure was quantified by gravimetrical analysis. ClearTip displayed a significantly lower uptake in comparison to Steripack, Puritan Flocked, and Puritan Foam control swabs. (**D**) Release quantification of injection molded, ClearTip, in comparison to commercially available controls in 2% w/v PEO loaded with 80% v/v FITC-labeled microparticles. *Significant effect of swab type (p < 0.05). Image partially created with Biorender.

To determine individual swab pick up, a gravimetric analysis was conducted. The model was loaded with 2% w/v PEO and allowed to saturate (Fig. 1A-B). Each type of swab (N = 5) collected a sample according to the swabbing procedure previously described. The dry swab and loaded swab were weighed and the difference was calculated. By measuring the difference, we estimated the mass of biological material collected by each swab in a clinically relevant swabbing workflow (Fig. 1C)*.* Average mass and standard deviation values are reported in Supplementary Table 1A. All the swabs studied displayed significant differences in terms of mass uptake (p < 0.05). The IM, nylon flocked, polyester flocked, and foam swabs are statistically different from one another in terms of mass uptake. ClearTip and Puritan Flocked demonstrated the minimum and maximum mass uptakes, respectively.

Swab release was quantified indirectly by using FITC labeled microparticles, as surrogate for cellular material, as previously reported^6^. The soft tissue portion of the model was saturated with the fluorescently labeled microparticles and was swabbed according to the procedure described above. The fluorescence was read and compared across swab types as cellular-mimicking release (Fig. 1D). Average fluorescence and standard deviation values are reported in Supplementary Table 1B. ClearTip swabs displayed a significantly greater release in cellular mimicking material in comparison to Steripack and Puritan Flocked swabs (p < 0.05). Conversely, Puritan Foam demonstrated a consistently greater release in comparison to all swab types (p < 0.05).

### Pooled swab release quantification

**Figure 2:**
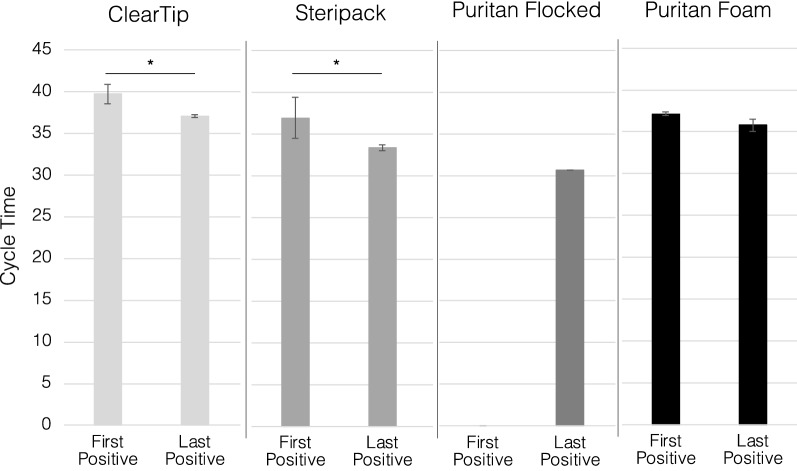
Swab performance in dip and discard pooling workflow (DDW). Quantification of swab performance by RT-qPCR quantification of N2 SARS-CoV-2 gene pickup DDW by varying the order of the positive swab in the pool of 10 swabs. Comparison of injection molded, ClearTip, Steripack, Puritan Flocked and Puritan Foam swabs validated in an anterior nasal tissue model loaded with healthy artificial mucus (negative sample) or spiked with heat-inactivated SARS-CoV-2 virus (positive sample). *Significant statistical difference (p < 0.05).

**Figure 3:**
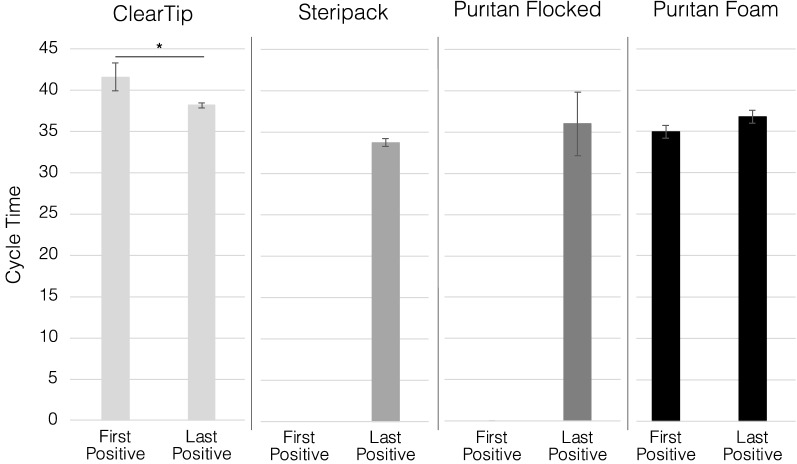
Swab performance in combine and cap pooling workflow (CCW). Quantification of swab performance by RT-qPCR quantification of N2 SARS-CoV-2 gene pickup CCW by varying the order of the positive swab in the pool of 10 swabs. Comparison of injection molded, ClearTip, Steripack, Puritan Flocked and Puritan Foam swabs validated in an anterior nasal tissue model loaded with healthy artificial mucus (negative sample) or spiked with heat-inactivated SARS-CoV-2 virus (positive sample). *Significant statistical difference (p < 0.05).

We investigated the effect of two different workflows for surveillance testing. The size of the pooling group (N = 10) was chosen upon preliminary studies (Data not reported) and usability investigations for surveillance testing^8^. The collection volume of 10 mL for all the studies was selected in comparison to 3 and 5 mL in preliminary investigations to allow the swab heads to be fully submerged in the viral transport medium for adequate release of biological material. To investigate the role of different absorbent and non-absorbent heads, relative volume retentions, and positive swab order in the pool of samples in the swab performance, we compared two different workflows: DDW and CCW (Figs. 2 and 3, respectively). DDW is based on sequentially dipping each swab in the sample vial and immediately discarding them at point of collection, while CCW dictates the collection and storage of all pooled samples, that are only discarded until they reach the CLIA-certified diagnostic lab. In order to assess the role of the positive sample order in the collection workflow, as swabs have different pick up and release characteristics, we analyzed the two extreme cases, a first and last positive swab in the pool of 10 samples. We then quantified the release of heat-inactivated viral material with RT-qPCR and expressed the results as Ct values.

In the DDW scenario (Fig. 2), both ClearTip and Steripack displayed a significant reduction in cycle time for last positive swab in comparison to first positive in the pool. Average Ct cycle threshold and standard deviation values are reported in Supplementary Table 2A. In the case of Puritan flocked, no viral material was detected in the first positive sample group, while the last positive displayed a signal above the 30th cycle. The positive sample order did not have an effect on the Puritan foam swab type with an average detection cycle of 35. In the CCW clinical case (Fig. 3), ClearTip consistently displayed a reduction in cycle time for the last positive swab workflow with detection values comparable to DDW. Average Ct cycle threshold and standard deviation values are reported in Supplementary Table 2B. On the contrary, the experimental group with Steripack swabs and a first positive sample order did not show any evidence of viral material detection, while the last positive sample group was comparable to DDW. Puritan flocked demonstrated a performance comparable to the DDW scenario, as detection signal was lost for the first positive sample group but displayed an increase in cycle time for the last positive sample detection group. The performance of Puritan foam swab was consistent in the CCW scenario, as reported for DDW, with no significant effects of workflow and positive sample order in viral detection.

### Volume loss quantification in pooling workflow

**Figure 4:**
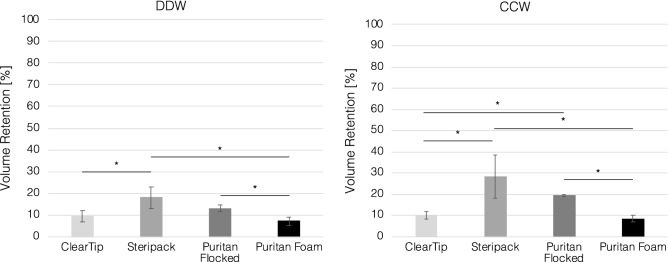
Swab volume retention in DDW and CCW pooling workflows. Quantification of volume retention in DDW and CCW pooling approaches for injection molded, ClearTip, Steripack, Puritan Flocked and Puritan Foam swabs. *Significant statistical difference (p < 0.05).

To understand difference in performance across swab types as a function of the different workflows investigated, we determined the volume loss of each pooled sample after all swabs were removed, as a direct effect of swab retention. After swab collection in 10 mL of 1 × PBS and vortexing, all swabs were discarded and the remaining volume of the pooled sample was measured, as reported in Fig. 4. For each swab type, we calculated the volume retention for each pooling workflow as percent of volume retention after all swabs have been discarded. Average volume retention and standard deviation values are reported in Supplementary Table 3A for DDW and Table 3B for CCW. There was no effect of collection workflow for both ClearTip™ and Puritan Foam, which also displayed comparable retention volumes. Both Steripack and Puritan Flocked demonstrated an increase in volume retention in CCW in comparison to DDW (p < 0.05), and greater retention in comparison to ClearTip and Puritan Foam, with Steripack displayed the greater volume retention across all swabs analyzed.

## Discussion

The use of a silk-glycerol sponge to mimic the soft tissue architecture of the nasal cavity and PEO to mimic the viscosity of native mucus are essential for the clinical relevance of this experimental validation tool. Previous studies have investigated uptake by dipping swabs into a vial of water or saline, which is not an accurate representation of clinical sample collection^9–11^. In our previous report, we demonstrated the relevancy and functionality of an in vitro nasal tissue model^6,7^ that allows replication of the clinical swabbing workflow with high fidelity, while being accessible to researchers, safe, reproducible, and time- and cost-effective. The precursor to the silk-glycerol sponge was a cellulose sponge that required large amounts of PEO to fully saturate it. We modified the model with a silk-based sponge to further mimic the mechanical properties of the nasal tissue, including the compressive modulus and saturation capability^12,13^. Silk has been used extensively in tissue engineering to replicate soft tissues which has made it a superior alternative to cellulose sponge^14–17^. The nasal model mimics the key architectural features of the anterior nasal cavity such as the diameter, length, and soft tissue properties, while incorporating an artificial mucus that mimics the viscosity of nasal fluid to accurately replicate the sample collection process^18^. In this experimental report, we aim to provide an experimental platform to mimic surveillance testing scenarios with mostly asymptomatic individuals. Since pooled testing is targeted at asymptomatic individuals, it is important to analyze fringe cases with low viral loads. The data presented here begins to characterize borderline pooling cases where variables such as swab type, workflow, and positive sample order can significantly affect the pooled PCR result. Despite drastically low Ct values presented by the delta variant^19^, we expect the Ct values of asymptomatic patients that participate in pooled testing to remain high, reinforcing the importance of studying the threshold cases defining positive and negative pools.

Individual swabs were characterized in terms of uptake and release before implementing them in pooled testing. We conducted pick-up quantification by swabbing the anterior nasal model saturated with artificial mucus and quantifying mass uptake by gravimetric analysis, which accurately mimics mucus collection. Our pick-up analysis indicated that the Yukon IM swabs picked up significantly less artificial mucus from the model in comparison to all other swab types. The Puritan nylon flocked swabs picked up 3.9 times more artificial mucus than the IM swabs. The Steripack polyester flocked swabs absorbed an even greater amount of mucus: 8.9 times more. The Puritan foam swabs picked up 2.5 times more artificial mucus than the IM swabs. In summary, gravimetric analysis determined that IM swabs pick up the least amount of mucus, while polyester flocked swabs picked up the most. Polyester flocked swabs, as seen in the gravimetric analysis, are the most absorbent of these four swab types. IM swabs represent the opposite end of the absorbance spectrum due to the grooved surface and hydrophobic properties, allowing to scoop and scrape viscous biological material without absorbing it, thus maximizing its release. On the contrary, the flocked and foam swab properties allow for maximum absorbance during pick-up, and potentially greater retention. Single swab release was quantified by spiking the artificial mucus with fluorescently labeled microparticles, as surrogate for cellular material^20^. Through this release quantification, fluorescence can be directly correlated to cellular particulates picked up and released by the swab. The IM swabs released significantly more microparticles than the polyester flocked swabs (1.6 times more particles) and slightly more than flocked swabs (1.1 times more particles). Foam swabs, however, appear to release the most material of all four swab types at 1.8 times more release than IM swabs. The pick-up and release quantification suggested that polyester flocked swabs exhibit the greatest sample retention, which aligns with the high absorbance of this swab type. In comparison, the IM swabs pick up the least amount of artificial mucus of all swab types and release the second largest amount of microparticles mimicking cellular material. This phenomenon is opposite of the polyester flocked swabs, suggesting that the IM swabs retain limited material which makes them a strong candidate for pooling when working with small sample volumes. Foam swabs demonstrated the greatest release by a relatively large margin, releasing about 1.8 times more material than the swab type with the second highest release (IM swabs). This analysis suggests that IM swabs would be optimal for collecting asymptomatic samples where the sample load is small, while the foam swabs would be preferable for symptomatic sample collection.

Differences in recovery of viral material between the two workflows (CCW and DDW) as a function of swab type was determined with RT-qPCR. The in vitro nasal model was loaded with heat inactivated SARS-CoV-2 spiked artificial mucus and swabbed according to the CDC collection guidelines^1^. CCW can be described as pooling all swabs simultaneously in transport media, vortexing, and discarding all the swabs in the CLIA-certified laboratory after transportation. DDW consists of dipping one swab in transport media, vortexing, and then removing the swab before adding the subsequent swab at the collection site. Due to the significant differences in swab absorbance, the order of the positive sample swab in the collection workflow also plays an important role in the identification of positive pools by high complexity molecular testing. When keeping the first swab positive, 3 out of the 4 swab types released sufficient viral material to be detected by RT-qPCR in DDW, while only in 2 out of the 4 swabs experimental groups were detected as positive in the CCW. This is due to the difference in volume retention of the swabs. When the last swab was positive, 4 out of 4 swabs were amplified in both workflows, but the CCW had an average Ct value of 1.9 higher than the DDW, due to the greater retention in the CCW workflow. These results demonstrated the critical role of the positive sample order in the collection workflow. Particularly, depending on the swab volume retention, it does significantly impact the detection of false negative pools in different pooling workflows. This is observed through the failed amplification of three positive pools when the positive sample was the first sample added to the pool. A total of 8 pools were conducted for each positive sample order (4 swab types and 2 workflows for each). 5 of these 8 pools showed amplification of viral material when the positive was added first, but virus was detected in all 8 pools when the positive sample was added last. The data presented here explains that positive sample order strongly influences positive pool detection and therefore should be considered when validating pooling procedures.

Important considerations can be drawn by analyzing differences between the DDW and CCW workflows. Across swab types and positive sample orders, CCW consistently presented a higher Ct value than DDW. This suggests that DDW is a more sensitive protocol in comparison to CCW. However, DDW requires the samples to be processed (i.e., vortexed, removed, and discarded) at the collection site, while CCW allows for samples to only be collected on site and then transported to a separate laboratory for sample processing. Thus, DDW presents increased risk exposure for personnel at the collection site due to aerosols generation during handling, in comparison to CCW. In summary, CCW demonstrated a reduced exposure risk during collection, while DDW has been shown to be more sensitive, depending on the swab type.

The difference in volume loss between workflows has been shown to be dependent on swab type. There was an average 32% increase in volume lost from DDW to CCW across all swab types. Nylon flocked, and polyester flocked swabs consistently displayed a greater volume loss in CCW than DDW, as reflected in increase of false negative pooling outcomes. In comparison, IM displayed the lowest volume reduction across all swab types, equivalent to only 10% of the initial volume. Polyester flocked and foam swabs showed a much larger increase in volume loss than IM swabs, at 50% and 40% respectively. Interestingly, foam swabs showed an opposite pattern, with more volume lost in DDW than CCW. In comparing overall swab performances, foam swabs defy the notion that absorbent swabs like polyester flocked pick-up more material at the expense of releasing less material when compared to unabsorbent swabs such as nylon flocked or IM. In our experiments, foam swabs both absorbed and released a relatively large amount of microparticles, which is ideal for sample collection. Additionally, foam swabs showed an opposite trend of volume loss in our two experimental workflows, as it is the only swab type that demonstrates increased volume loss in DDW. Together, this information suggests the presence of an underlying mechanism that is unique to foam swabs. Polyurethane foams have been shown to behave in a viscoelastic fashion and experience stress relaxation^21^. Also, one group has shown that the Young’s modulus of polyurethane foams decreases with water absorption^22^. Based on this data and our observation of foam swabs, we hypothesize that stress relaxation is responsible for these differences between foam and other swab types. As a foam swab absorbs sample, the tip swells and experiences stress due to the retained fluid. When applied over a longer period, as seen in CCW, this stress/relaxation phenomenon may induce an increase in pore size that causes the swab to become less absorbent and release more material.

The data presented here highlights the impact of swab type, positive sample order, and workflow when conducting pooled testing for COVID-19 and justifies the consideration of these variables in future pooling efforts. Future work could include additional variables, such as soak time, vortex time, varying viral loads, and clinical trials with known positive patients.

## Methods and materials

### 3D anterior nasal tissue model preparation

**Figure 5:**
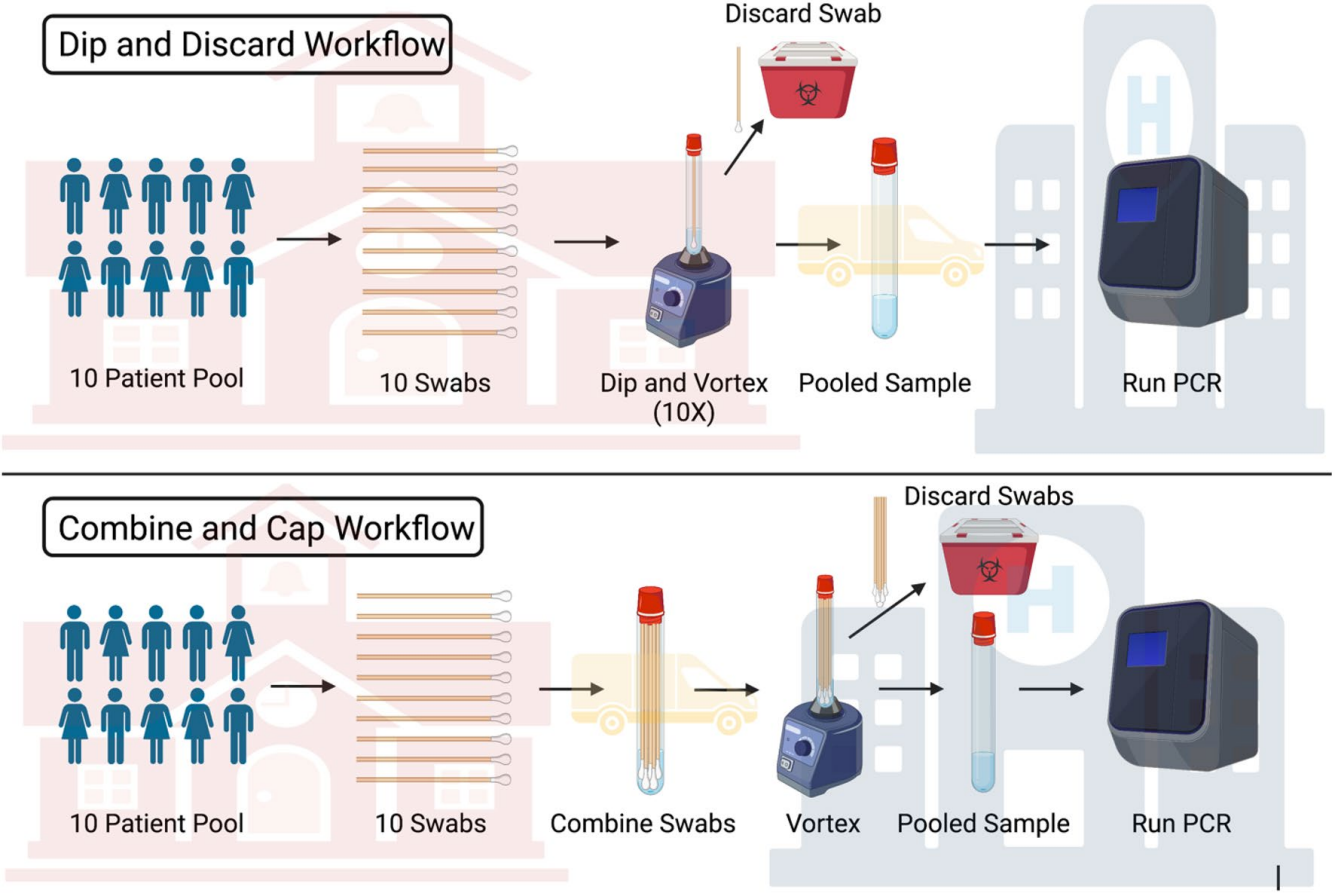
Workflow overview: we propose two distinct pooling workflows: dip and discard workflows (DDW) and combine and cap workflow (CCW). Both protocols describe a combination of samples which eliminates dilution associated with combination of aliquots. DDW involves collection of swab samples from a group of patients, sequentially adding each swab to a vial of transfer media, vortexing, discarding the swab, and repeating these steps for each swab sample. The pooled sample in transfer media is then transported to a CLIA certified lab for PCR testing. CCW follows similar steps, with the difference being the swab samples are added simultaneously to the transfer media without vortexing before being sent to a CLIA lab. Created with Biorender.

**Figure 6:**
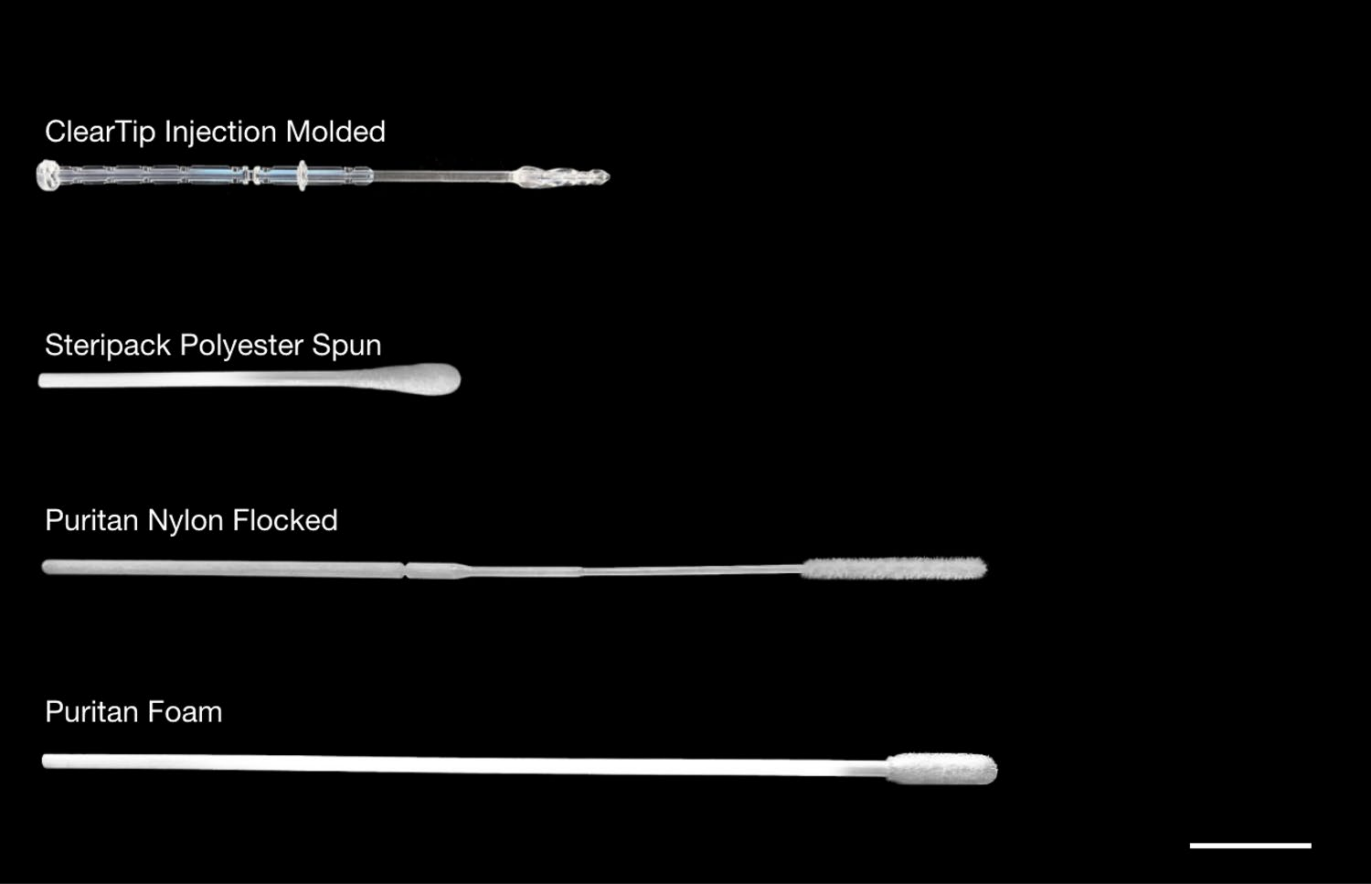
Macro images of anterior nasal experimental swabs. Four swabs were used in this study, from top to bottom: ClearTip Injection Molded swab, Steripack Polyester Flocked swab, Puritan Nylon Flocked swab, and Puritan Foam swab. Scale bar = 20 mm.

Based on our previously developed 3D nasal cavity tissue model^6,7^, we utilized a bench top validation system to study different pooling workflows (Fig. 5) and relative swab performance (Fig. 6). The 3D nasal tissue model was comprised of a silk-based sponge saturated with a mucus-mimicking solution (Fig. 1A). The anterior nasal tissue model is composed of a 1 mm thick silk-glycerol sponge to mimic the soft tissue nature of the nasal cavity. To confine and retain the mucus, the tissue model was inserted in a 1.5 cm long silicone tubing to mimic the depth the swab needs to be inserted according to the CDC (Fig. 1B)^23^. The final inner diameter was 10 mm to mimic the average diameter of the human nostril^24^. To mimic the soft tissue of the nasal cavities, aqueous silk sponges were prepared as previously reported^12,25^. Briefly, pure silk fibroin was extracted from *Bombyx mori* cocoons by degumming the fibers in a sodium carbonate solution (0.02 M) (Sigma-Aldrich, St. Louis, MO) for 30 min to remove sericin. The degummed fibers were rinsed three times and dried overnight before the solubilization in 9.3 M lithium bromide (Sigma-Aldrich, St. Louis, MO) for 2 h at 60 °C. The obtained solution was dialyzed for 3 days against DI water using a standard grade regenerated cellulose dialysis tubing (3.5 kDa MWCO, Spectrum Labs Inc, Rancho Dominguez, CA). The solution was then centrifuged to remove impurities. The silk-based sponge structure was prepared as previously described^12^. Briefly, silk aqueous solution (6% wt/vol) was mixed to achieve a 30 wt% glycerol solution and then poured into a rectangular casting vessel and dried in a laminar flow hood at room temperature for 48 h to achieve formation of a film substrate. A 6% wt/vol silk aqueous solution and 30 wt% glycerol was then mixed with sieved granular NaCl (500–600 μm, average crystal size) in a ratio of 2 g NaCl per mL of silk fibroin solution and layered on to the surface of the film. The resultant solution was allowed to cast and fuse to the silk film for 24 h at 37 °C and NaCl and 3 days at room temperature. The salt was subsequently removed by washing the scaffold for 72 h in distilled water with regular volume changes. The sponges were autoclaved at 121 °C and allowed to dry in a biological hood before loading with mucus mimicking solution. The mucus mimicking solution was prepared from a 2 wt% polyethylene oxide (PEO) solution (Sigma-Aldrich, St. Louis, MO. MW 100,000–200,000), that was previously shown to have similar viscosities to healthy nasal mucus^26^. The anterior nasal tissue model was then saturated with 1.875 mL of the physiologically relevant mucus solution, and re-saturated with 100 μL after each swab.

### Experimental swabs

These experiments were conducted using four different types of swabs: injection molded (IM) (ClearTip—Yukon Medical, Durham, NC), polyester flocked (Polyester Spun Swab—SteriPack—Lakeland, FL), nylon flocked (PurFlock—Puritan, Guildford, ME), and a foam tipped (Foam Tipped Applicator—Puritan, Guilford, ME), reported in Fig. 6. None of the swabs were directly from patients or from a pathology lab.

### Single swab pick up quantification

To quantify swab uptake, the anterior nasal tissue model was saturated with the physiologically relevant artificial mucus solution^6,7^ and the following swabbing procedure was utilized. Each swab was inserted into the model, twisted around the nasal model surfaces five times, held in place for 15 s, and then removed. The pick-up swab quantification was performed by gravimetric analysis, and the weight of each swab (N = 5) was recorded before and after the swabbing procedure and reported as mass uptake. This was performed for each of the four swab types.

### Single swab release quantification

In order to efficiently assess cellular material uptake, the model was saturated with 10 μm fluorescently labeled microparticles to mimic cellular particulates into the artificial nasal solution, as previously reported^6^. FITC-labeled microparticles (Sigma-Aldrich, St. Louis, MO), based on melamine resin, were dispensed into the tissue model, and allowed to saturate the silk sponges. The above-mentioned swabbing procedure was performed. Each swab (N = 5) was then removed and placed in 1 mL volume of phosphate buffer solution (PBS 1 ×) (VWR Scientific, Radnor, PA). 100 μL aliquots were taken in triplicate and analyzed with a SpectraMax M2 plate reader for fluorescence at 525 nm emission and 490 nm excitation. After background subtraction, fluorescence signal was then reported as an expression of cellular-mimicking uptake.

### Pooling workflows

We investigated several experimental variables that could influence the outcome of pooled testing: swab type, swab processing workflow, and positive sample order. The two different workflows are described in Fig. 5. The Dip and Discard Workflow (DDW) is described as swabbing the patient and transferring the swab into the collection vial with a selected transport media (Viral Transport Media, PBS, etc.). The vial is then vortexed for 15 s and the swab discarded at the collection site. The subsequent swab from the next patient is then placed in the same tube and transport media as the previous swab, vortexed for 15 s, and the swab is discarded. This procedure is repeated for 10 swabs, with the first or last swab being positive, while the remaining swabs are negative. The collection vial is then shipped to a Clinical Laboratory Improvement Amendments (CLIA)-certified lab for analysis with RT-qPCR. In comparison, the Combine and Cap Workflow (CCW) consists of collecting 10 separate swab samples and placing them all into one collection vial with a selected transport media. The collection vial will be shipped to a CLIA-certified lab, where the tube is vortexed for 15 s, the swabs are then discarded, and the pooled samples analyzed with RT-qPCR.

### Positive sample order

In practice, a positive sample could be added at any point during the workflow. We compare the impact of positive sample first and positive sample last on each workflow, which covers both extremes of possible positive sample order. We established two models, one positive model with SARS-CoV-2 spiked artificial mucus, and one negative model with plain artificial mucus. The positive model was either swabbed first or last for each workflow and for each swab type, while the remaining swabs were used to collect sample from the negative model. The resulting Ct values from pools with different combinations of workflow and positive sample order were analyzed to determine the impact of these variables on pooling sensitivity.

### Volume loss quantification

Volume loss after swab collection in both workflows was measured. During both workflows, swabs are added to and removed from a collection tube containing 10 mL of PBS. The swabs uptake some of the PBS when removed, decreasing the remaining volume in the tube. The remaining volume was measured, and the difference was calculated and expressed as a percent of the initial volume.

### Pooled swab release quantification

To study the effect of the different pooling variables on viral detection, the nasal model was saturated with the nasal solution spiked with 10^7^ copies/mL of heat-inactivated SARS-CoV-2, USA-WA1/2020 (NR-52286, BEI Resources, ATCC, USA), and the swabbing procedure was carried out, as described above. 10 swabs for each type were then collected in a collection vial with 10 mL of PBS, following the two workflows described above. We also explored the cases of the first and last positive swabs and their relative impact. 5 µL from each sample was then tested to quantify the detection of SARS-CoV-2. To detect presence of SARS-CoV-2, we carried out the CDC 2019-Novel Coronavirus (2019-nCoV) Real-Time RT-PCR Diagnostic Panel (https://www.fda.gov/media/134922/download), per manufacturer instructions using the 2019-nCoV_N1 Combined Primer/Probe Mix with a Quantabio Ultraplex One-Step RT-qPCR ToughMix, as previously reported^7,27^. Gene amplification was carried out accordingly to manufacturer instructions with a QuantStudio™ 5 Real-Time PCR System (Thermo Fisher Scientific, Waltham, MA, USA). The cycle threshold (Ct) value was reported for each swab (N = 5).

### Statistical analysis

Statistical analysis was performed using a Analysis of Variance (ANOVA) single factor with a p-value of < 0.05 using Origin (Pro), Version 2021b OriginLab Corporation, Northampton, MA, USA.

## Conclusions

Testing during the COVID-19 pandemic faced countless limitations, including global shortage of diagnostic reagents. By pooling patient swab samples, surveillance bandwidth can be significantly increased without consuming additional testing resources or sacrificing sensitivity. To limit false negative pooling outcomes, swab type, workflow, and positive swab order effects on RT-qPCR, media volume loss, and swab pick up and release were investigated. An anterior nasal cavity based on silk sponges to mimic the soft tissue of the cavity was used to recreate clinical workflows on the bench to accelerate swab device validation at reduced experimental expenses associated with clinical patient samples. We conclude that swab type, workflow, and positive sample order have a significant impact on the sensitivity of detection of SARS-CoV-2 in pooled samples, and therefore must be considered when using pooled samples to conduct screening for COVID-19.

## Supplementary Information

Supplementary Tables.

## Data Availability

The data that support the findings of this study are available from the corresponding author, C.E.G., upon reasonable request.
