# Supplementary material for: Underscoring the effect of swab type, workflow, and positive sample order on swab pooling for COVID-19 surveillance testing
Source: Sci Rep. 2023 May 3;13:7174. doi: 10.1038/s41598-023-34337-y (PMC10155136; doi:10.1038/s41598-023-34337-y)
Supplement: Supplementary file 1 — Supplementary Tables. [file 41598_2023_34337_MOESM1_ESM.docx]

**Supplementary Material**

| **ClearTip** | **Steripack** | **Puritan Flocked** | **Puritan Foam** |
| --- | --- | --- | --- |
| 19.00$\pm$4.96 | 169.98$\pm$35.72 | 74.3$\pm$12.63 | 47.38$\pm$11.44 |

**Supplementary Table 1a: Pick Up Quantification Data.** Average $\pm$ standard deviation (mg) of swab pick up.

| **ClearTip** | **Steripack** | **Puritan Flocked** | **Puritan Foam** |
| --- | --- | --- | --- |
| 100.00$\pm$8.18 | 61.93$\pm$9.69 | 90.66$\pm$17.49 | 177.74$\pm$25.29 |

**Supplementary Table 1b: Release Quantification Data**. Average $\pm$ standard deviation (Fluorescence %) of swab release.

| **ClearTip** | | **Steripack** | | **Puritan Flocked** | | **Puritan Foam** | |
| --- | --- | --- | --- | --- | --- | --- | --- |
| First | Last | First | Last | First | Last | First | Last |
| 39.71$\pm$1.16 | 37.03$\pm$0.17 | 36.95$\pm$2.47 | 33.37$\pm$0.33 | / | 30.66 | 37.12$\pm$0.22 | 35.80$\pm$0.72 |

**Supplementary Table 2a: Swab Performance in Dip and Discard Workflow.** Average $\pm$ standard deviation RT-qPCR cycle threshold (Ct) values by varying positive swab order in the DDW.

| **ClearTip** | | **Steripack** | | **Puritan Flocked** | | **Puritan Foam** | |
| --- | --- | --- | --- | --- | --- | --- | --- |
| First | Last | First | Last | First | Last | First | Last |
| 41.58 $\pm$1.67 | 38.15$\pm$0.32 | / | 33.75$\pm0.44$ | / | 35.98$\pm$3.81 | 34.93$\pm$0.78 | 36.75$\pm$0.76 |

**Supplementary Table 2b: Swab Performance in Combine and Cap Workflow (CCW).** Average $\pm$ standard deviation RT-qPCR cycle threshold (Ct) values by varying positive swab order in the CCW.

| **ClearTip** | **Steripack** | **Puritan Flocked** | **Puritan Foam** |
| --- | --- | --- | --- |
| 10 $\pm$2.45 | 19.2 $\pm$ 5.02 | 13.1 $\pm$ 1.56 | 7.17 $\pm$2.02 |

**Supplementary Table 3a: Swab Volume Retention in Dip and Discard Workflow (DDW).** Average $\pm$ standard deviation (Volume Retention %) of swab volume retention in DDW.

| **ClearTip** | **Steripack** | **Puritan Flocked** | **Puritan Foam** |
| --- | --- | --- | --- |
| 10.10 $\pm$ 1.76 | 28.33 $\pm$10.21 | 19.27 $\pm$0.40 | 8.33 $\pm1.52$ |

**Supplementary Table 3b: Swab Volume Retention in Combine and Cap Workflow (CCW).** Average $\pm$ standard deviation (Volume Retention %) of swab volume retention in DDW.
